# Analysis of breeding efficiency and application of insect-resistant and herbicide-tolerant maize LD05

**DOI:** 10.1080/21645698.2026.2753273

**Published:** 2026-11-11

**Authors:** Xinwei Hou, Wenlan Li, Guoqi Yao, Zhaodong Meng, Zhaohua Ding, Runqing Yue

**Affiliations:** Maize Research Institute, Shandong Academy of Agricultural Sciences, National Engineering Research Center of Wheat and Maize, Shandong Key Laboratory of Maize Biological Breeding, Jinan, China

**Keywords:** Breeding efficiency, field trial, GM maize LD05, herbicide tolerance, lepidopteran insect resistance

## Abstract

Maize is one of the most important grain crops in China and suffers severe damage from lepidopteran pests, with annual yield losses reaching 15–20%. Long‑term reliance on chemical pesticides has led to increasing problems of resistance, environmental pollution, and food safety. Transgenic insect‑resistant maize offers a new approach for green pest management. Although insect-resistant and herbicide-tolerant maize LD05 carrying the fusion gene *m2cryAb‑vip3A* has been granted a biosafety certificate, its breeding efficiency and performance in elite maize hybrids have not been systematically investigated. In this study, the *m2cryAb‑vip3A* and *bar* genes from LD05 were successfully introgressed into two elite inbred lines, Qixi5101 and DH382, through backcrossing combined with marker‑assisted selection. Compared with LCV01, which carries two independent insect‑resistant genes, the fusion gene strategy significantly improved breeding efficiency. The genetically modified (GM) inbred lines exhibited high resistance to lepidopteran pests and tolerated four times the recommended medium dose of glufosinate. Developed from these GM inbred lines, the GM hybrids Ludan509 and Denghai605 maintained original yield levels while exhibiting excellent insect resistance and herbicide tolerance, thereby reducing field management costs and increasing economic benefits. This study presents the complete pre‑commercialization chain of GM maize, from LD05 transformation to hybrid combination and field validation, providing an efficient, simple and cost-effective technical pathway for multi-trait improvement. This strategy provides a practical and efficient alternative for multi-trait improvement in crop breeding and offers technical support for the sustainable development of modern agriculture.

## Introduction

Maize is one of the most important food crops in the world, serving as grain, feed, and industrial raw material, and it occupies a central position in ensuring national food security.^1,2^ Maize production is chronically and severely damaged by lepidopteran pests such as *Ostrinia furnacalis* and *Spodoptera frugiperda* all year round, which not only cause substantial yield losses and quality deterioration but also significantly increase production inputs and control costs.^3,4^ Annual losses of maize yield due to insect pests can reach 10%–20%.^5,6^ Furthermore, weed competition in the field further exacerbates yield losses, making integrated pest management a critical issue that urgently needs to be addressed.^7,8^ For a long time, chemical pesticides have been the primary means of controlling maize pests and weeds; however, over-reliance on chemical control has led to the continuous evolution of pest resistance, increasingly severe environmental pollution, and escalating risks to agricultural product safety, rendering traditional control models unsustainable.^9–11^ In contrast, the development of transgenic insect-resistant and herbicide-tolerant maize provides a new pathway for green pest management.^12^ By continuously expressing *Bacillus thuringiensis* (Bt) proteins, such maize kills target pests and reduces the application of chemical pesticides, while also enabling field weed management through herbicide tolerance. This approach safeguards yield while reducing adverse impacts on the ecological environment and agricultural product safety.

GM insect-resistant maize expressing Bt protein provides an effective strategy for pest management.^13^ Since the first commercial cultivation of GM maize in 1996,^14,15^ a total of over 244 GM maize transformation events have been approved globally, of which 210 contain insect-resistant traits, fully demonstrating the central role of insect resistance traits in GM maize research and development.^2,3^ Major international seed companies such as Monsanto (now part of Bayer) and DuPont Pioneer (now part of Corteva) have successfully developed a series of insect-resistant maize varieties and gene-stacked varieties, including MON810, TC1507, and MIR162, effectively controlling the damage by lepidopteran pests such as *O. furnacalis* and *S. frugiperda*, and significantly improving economic, social, and ecological benefits.^3^ In recent years, China has initiated pilot planting of multiple GM maize varieties. By 2024, the control efficacy of these GM varieties against lepidopteran pests had exceeded 90%.^16^ In 2025, 96 insect-resistant herbicide-tolerant GM maize varieties were approved by the Ministry of Agriculture and Rural Affairs (Ministry of Agriculture of the PRC (https://www.moa.gov.cn/)). These GM maize varieties exhibit strong control capabilities against *O. furnacalis* and *S. frugiperda*, laying a solid foundation for commercial application. However, large-scale planting of GM insect-resistant maize has accelerated the evolution of pest resistance to Bt protein.^17–21^ At present, several lepidopteran pests (especially *O. furnacalis* and *S. frugiperda*) have developed resistance to various Cry proteins (Cry1F and Cry1Ab), posing a threat to the sustainable application of GM maize.^22^ In response to this increasingly severe situation, global efforts have focused on developing GM maize events that simultaneously express multiple Bt proteins (such as Cry1, Cry2, Vip3Aa) through multigenic insect resistance strategies.^4,23–25^ This strategy not only broadens the insecticidal spectrum but also helps delay the evolution of pest resistance.

Currently, there are two main technical approaches for achieving multi-gene insect resistance: polygene polymerization^23,26^ and fusion genes.^27,28^ Polygene polymerization stacks multiple independent genes via hybrid breeding, offering independent expression and ease of marker selection but suffers from long breeding cycles, linkage drag, and high regulatory costs. The fusion gene approach links multiple coding sequences into a single open reading frame driven by one promoter, enabling co‑expression from a single event with simpler inheritance and lower approval costs, though it carries potential risks such as complex protein folding or steric hindrance. International seed companies generally adopt gene stacking of *Cry* and *Vip3Aa* as the research direction for next-generation insect-resistant maize.^29^ Unlike the predominant approach of gene stacking through crossbreeding both domestically and internationally, GM maize LD05 was transferred with the insect resistance fusion gene *m2cryAb-vip3A* with independent property rights.^30^ This gene is a novel insect-resistant fusion gene created by combining sequence- and structure-optimized fragments of *cry1Ab* and *vip3Aa*, achieving the innovation of an insect resistance gene with two protein activities, Cry1Ab and Vip3Aa, thereby expanding the insecticidal spectrum and enhancing insect resistance.

The successful introgression of transgenic traits into elite germplasm is a key step in realizing the application potential of transgenic crops. Merely obtaining a transgenic event is not sufficient to achieve this goal; the target traits must also be efficiently introgressed into elite varieties with good local adaptation and high yield potential so that they can truly translate into significant benefits for agricultural production.^12,14,31,32^ In a 3-y trial in northeastern China, transgenic insect-resistant and herbicide-tolerant maize achieved over 99% control of locally prevailing lepidopteran pests and 97% weed control, increased grain yield by 4.6%–10.1%, reduced mycotoxin contamination by 91.7%–95.6%, and increased farmers’ net income by US$276–332 per hectare under subsidized seed prices.^12^ In this study, two previously developed transformation events, LD05 and LCV01, which were generated via the fusion gene and polygene polymerization strategies, respectively, were selected for investigation. Using backcrossing combined with marker-assisted selection, the target traits from LD05 and LCV01 (containing two independent insect-resistant genes *Cry1Ab* and *Vip3Aa*) were introgressed into two elite maize inbred lines, Qixi5101 and DH382. Qixi5101 (LD05) and DH382 (LD05) exhibit high insect resistance across multiple generations. The Ludan509 (LD05) and Denghai605 (LD05), derived from two GM inbred lines, retained their original agronomic characteristics while showing excellent insect resistance and herbicide tolerance. This study provides practical evidence for the efficient application of the fusion gene strategy in maize breeding, validating a feasible pathway from transformation event to commercial variety.

## Materials and Methods

### Plant Materials

LD05 and LCV01 were developed by the Shandong Academy of Agricultural Sciences. LD05 contains a fusion insect-resistant gene, *m2cryAb-vip3A*, which is linked with a glufosinate resistance gene. LCV01 contains two independent insect-resistant genes, *Cry1Ab* and *Vip3Aa*, which are respectively linked with a glyphosate resistance gene and a glufosinate resistance gene. The non-GM materials Qixi5101 and Ludan509 were preserved by the Shandong Academy of Agricultural Sciences. The non-GM DH382 and Denghai605 were provided by Shandong Denghai Seeds Co., Ltd. The experiments were conducted at the artificial climate chamber of the Maize Research Institute, Shandong Academy of Agricultural Sciences; the Huang-Huai-Hai Transgenic Maize Pilot and Industrialization Base at Dangjia Village, Longshan Subdistrict, Zhangqiu District, Jinan City, Shandong Province; and the Phase II Experimental Base of the National Southern Breeding Biological Breeding Special Zone at Yazhou District, Sanya City, Hainan Province. Zhangqiu District is located in the transition zone between the central Shandong hills and the Yellow River alluvial plain, featuring a warm‑temperate semi‑humid monsoon climate. Summer maize is generally sown in mid‑June and harvested from late September to early October, with a total growth period of approximately 100‑110 d. The major insect pests encountered during the entire growth period of maize in this region include *Ostrinia furnacalis*, *Helicoverpa armigera*, and *Mythimna separata*. Yazhou District is located in the southern coastal area of Hainan Island, characterized by a tropical oceanic monsoon climate with year-round summer conditions and distinct wet and dry seasons. Maize is sown in early October and harvested from late January to early February of the following year, with a total growth period of approximately 110–120 d. The major insect pests of maize in this region include *Spodoptera frugiperda*, *Ostrinia furnacalis*, *Helicoverpa armigera*, and *Mythimna separata*. Uniform agronomic practices for maize cultivation, including fertilization, irrigation, and weed management, were implemented according to local conditions in each region. During the field experiments for insect resistance and herbicide tolerance, insecticides and herbicides were applied according to the requirements of each experiment.

#### Screening and Development of GM Inbred Lines and Hybrids

LD05 and LCV01 were each crossed with the recurrent parents Qixi5101 and DH382 to generate F_1_ hybrids. From the BC_1_F_1_ generation onward, herbicide application was first used to screen for herbicide tolerance in each generation: LD05 populations were sprayed with glufosinate at 1× the field-recommended medium dose, whereas LCV01 populations were sprayed with both glufosinate and glyphosate. Only plants that survived herbicide application were subsequently subjected to two to three independent PCR assays to confirm the presence of the target genes. Selected positive plants were backcrossed to the recurrent parent to produce the next backcross generation. This process was repeated until the BC_8_F_1_ generation. BC_8_F_1_ plants were self-pollinated for two consecutive generations (B_8_F_2_ to BC_8_F_3_) to obtain homozygous lines. In every generation, only plants confirmed positive by both herbicide screening and PCR were used for further crossing or selfing. Because LD05 carries a fusion gene at a single locus, the theoretical positive rate in its backcross progeny is approximately 50%, twice that of LCV01 (approximately 25%), which carries two independent loci. Therefore, in practical backcross screening, 300 plants per generation were planted for the LD05 population, while 600 plants per generation were planted for the LCV01 population. In subsequent selfing generations, the homozygous positive rate of the target gene after selfing of the LD05 heterozygote was 25%, whereas the probability of simultaneous homozygosity at the two independent loci in LCV01 was only 6.25%, meaning the former was four times the latter. Accordingly, in selfing screening, 300 plants per generation were planted for the LD05 population, while 1200 plants per generation were required for the LCV01 population to ensure that the LCV01 population yielded the same number of homozygous plants as the LD05 population. At the BC_8_F_1_ generation, a developed 5K liquid‑phase chip was used to detect the background recovery rate of the positive plants. Those with a high background recovery rate were selfed for three consecutive generations for creating the GM hybrids Ludan509 (LD05) and Denghai605 (LD05).

#### Gene and Protein Expression Levels in Different Maize Tissues

To examine the stability of target gene transcription and translation in GM materials, Ludan509 (LD05) and Denghai605 (LD05) were planted. Inter-row and intra-row plant spacing were 0.5 and 0.2 m, respectively. Standard cultivation practices were followed. Tissues were collected at the V5 (roots, stems, leaves), silking (silks, pollen), and mature (kernels, bracts) stages. For each sample, three plants were pooled as one replicate, with three replicates in total. All tissue samples were flash‑frozen in liquid nitrogen and stored at −80°C.

Total RNA was extracted by using a total RNA extraction kit (Tiangen Biotech (Beijing) Co., Ltd.). cDNA synthesis and purification were performed according to the manufacturer’s instructions of the reverse transcription kit. Three technical replicates were performed for each sample. The average Ct values of the reference gene (*zSS b11*) and the target gene in each sample were detected by qRT-PCR. The expression levels of the target gene in the sample were calculated using the formula RQ = 2^(-∆Ct). These results were statistically analyzed and presented as mean ± SD.

The M2cryAb-vip3A protein ELISA kit was developed by Wuhan Laikenboao Technology Co., Ltd., and the PAT protein ELISA kit is a commercial kit. The kits were equilibrated at room temperature for 30 min, and all reagents were shaken well before use. All procedures were performed according to the manufacturer’s protocols, and each sample was assayed in three technical replicates. The procedure included diluting the standard, adding samples/standards, incubating at room temperature in the dark, washing the plate, adding enzyme-labeled working solution, repeating the washing, color development, and measurement. The absorbance at OD450 nm was measured for each well using a microplate reader set at dual wavelengths (450/630 nm).

#### Bioassays with Different Plant Tissues

In 2020, populations of *O. furnacalis*, *M. separata*, *H. armigera*, and *S. frugiperda* were collected in Guangdong Province. All tested insects were reared in the laboratory for more than two generations to ensure a relatively consistent genetic background, and the previous two generations had not been exposed to any chemical pesticides or insect-resistant GM materials. Leaves (V5 stage) and silks (silking stage) were collected from LD05, LCV01, BC_8_F_3_–BC_8_F_5_ generations of Qixi5101 (LD05) and DH382 (LD05), Ludan509 (LD05), and Denghai605 (LD05); kernels (mature stage) were collected only from the two GM hybrids. Twenty target tissue samples (leaves, silks, or kernels) were randomly taken from 20 maize plants. Leaves and silks were placed on rearing plates, with 20 replicates per tissue sample, and each tissue sample was infested with 5 neonate larvae. Kernels were placed into well‑type rearing trays (one kernel per well), and each well was infested with one neonate larva, with a total of 20 replicates. Larvae were reared in an insectary at 26–28°C, with a 16 h:8 h light:dark photoperiod and a relative humidity of 70–80%. Fresh plant tissue was replenished daily according to larval consumption. Larval survival was recorded daily for 5 consecutive days; a larva was considered dead if it showed no response to stimulation with a fine brush or exhibited obvious growth inhibition.

The mortality rate was calculated using the formula:

$$x=\frac{N-n}{N}\times 100\%,$$

Where x is the mortality rate, n is the number of surviving larvae, and n is the number of infested larvae.

The corrected mortality rate was calculated using the formula:

$$y=\frac{x_{t}-x_{0}}{1-x_{0}}\times 100\%,$$

Where y is the corrected mortality rate, $x_{t}$ is the mortality rate on the test material, and $x_{0}$ is the mortality rate on the susceptible control material.

#### Field Trial

To evaluate the insect resistance of GM maize materials (including LD05, LCV01, BC_8_F_3_-BC_8_F_5_ generations of Qixi5101 (LD05) and DH382 (LD05), Ludan509 (LD05), and Denghai605 (LD05)) under field conditions, a randomized complete block design was used, with three replicates per treatment. Each plot had an area of 30 m^2^ and consisted of five rows (with a row spacing of 60 cm and a plant spacing of 25 cm). The interval between different pest inoculation plots was 2 m to avoid the spread of pests between plots. Border rows were planted around the experimental field to minimize edge effects. At the V5 stage, 30 neonate larvae of *Ostrinia furnacalis*, *Mythimna separata*, *Helicoverpa armigera*, or *Spodoptera frugiperda* were individually inoculated into the whorl of each plant using a fine brush, after which the whorl was covered with a mesh bag to prevent larval escape and cross-plot contamination. At the silking stage, 30 neonate larvae were placed directly onto fresh silks and the ear was covered with a paper bag. Each growth stage was inoculated twice, with an interval of 3 d between inoculations. For each treatment at each stage, no fewer than 40 plants were artificially infested. At 14–21 d after insect exposure at the V5 stage, leaf feeding damage on the upper and middle leaves of each plant was assessed individually. At 14–21 d after inoculation at the silking stage, ear and stem damage was assessed. According to the Ministry of Agriculture of the People’s Republic of China Announcement No. 953–10.1–2007, Environmental Safety Testing of Genetically Modified Plants and Their Products—Insect-Resistant Corn—Part 1: Insect Resistance, the resistance levels of the materials to target pests were classified based on the leaf feeding rating and the damage rating of ears and stems.

#### Analysis of Glufosinate Resistance

To evaluate the Glufosinate resistance of GM maize materials (Qixi5101 (LD05), DH382 (LD05), Ludan509 (LD05), and Denghai605 (LD05)). The field trial was arranged in a randomized complete block design with three replications. Each plot measured 30 m^2^ and consisted of five rows, with a row spacing of 60 cm and a plant spacing of 25 cm. Plots were separated by 1 m bare-soil alleyways. When the GM inbred lines, GM hybrids, and non-GM controls grew to the V5 stage, Glufosinate was sprayed onto all leaves. The herbicide application doses were: water (0×), 1× the field-recommended medium dose (1×, 3 L·hm^−2^), 2× (6 L·hm^−2^), and 4× (12 L·hm^−2^). The herbicide was sprayed in the late afternoon on a calm, rain-free day. Survival rate and damage rate were assessed at 1, 2 and 4 weeks after herbicide application. The experimental design and investigation methods followed the “Environmental Safety Testing of Transgenic Plants and Their Products – Herbicide-Resistant Maize – Part 1: Herbicide Tolerance” (Ministry of Agriculture and Rural Affairs Announcement No. 953–11.1–2007).

#### Benefit Investigation and Maize Yield Evaluation

To comprehensively evaluate the cost and benefit of GM hybrids, 3‑y field trials (2023, 2024, and 2025) were conducted at Jinan and Hainan, respectively. A randomized complete block design was used, with three plots per hybrid. Each plot had an area of 300 m^2^ (row spacing 60 cm, plant spacing 25 cm). The diagonal three‑point sampling method was adopted, with 100 plants sampled at each point. Pest incidence was recorded at five growth stages: V6, Vt, R1, R3, and R5. All ears from the three sampling points in each plot were harvested to calculate yield. Additionally, the costs of insecticide and herbicide applications during field management were recorded.

#### Statistics and Analysis

For data meeting the assumptions of normality and homoscedasticity, one-way ANOVA or two-sample t-test was applied; for data violating normality and homoscedasticity, a nonparametric Kruskal–Wallis test followed by pairwise Mann–Whitney U-test was used. All data analyses were performed using SPSS 20.0.

## Results

### Identification of Insect Resistance of LD05 and LCV01

To identify the resistance of LD05 and LCV01 to *O. furnacalis*, *M. separata*, *H. armigera*, and *S. frugiperda*, the leaves of LD05 and LCV01 at the V5 stage were collected for laboratory bioassays. At 3 d post-infestation, the corrected mortality rates of *O. furnacalis*, *M. separata*, *H. armigera*, and *S. frugiperda* were 100.00 ± 0.00% on leaves of both LD05 and LCV01 Figure 1(A-D). To evaluate the resistance performance of GM maize LD05 and LCV01 in the field, leaves at the V5 stage and silks at the silking stage were inoculated with target pests according to the lepidopteran species that naturally occur in different growth stages of maize. The leaf and silk damage levels of LD05 and LCV01 were significantly lower than those of non-GM maize Zheng58 Figure 1(E). These results indicate that LD05 and LCV01 have strong resistance against *O. furnacalis*, *M. separata*, *H. armigera*, and *S. frugiperda*.

**Figure 1.**
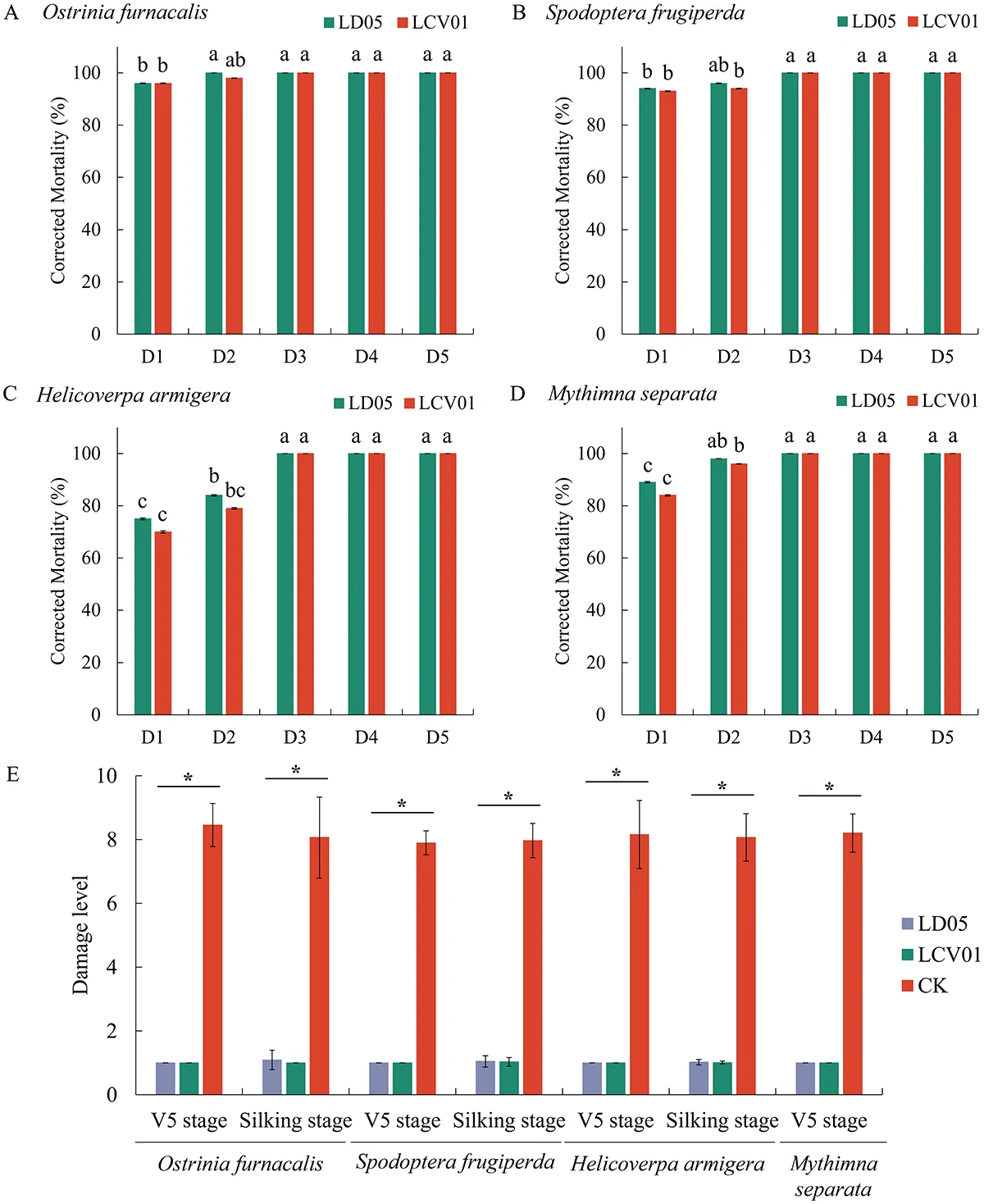
Identification of the resistance of LD05 and LCV01 to target pests. A-D: corrected mortality from 1–5 d post-infestation for four target pests. E: damage levels of four target pests at V5 and silking stage in field, respectively. Data are presented as mean ± standard error (*n* = 20 technical replicates per sample). Different letters and asterisks above the error bars indicate significant differences in corrected mortality at different time points (*p* < .05). ck: Zheng58; D1: day 1; D2: day 2; D3: day 3; D4: day 4; D5: day 5.

#### Analysis of LD05 Breeding Efficiency

To compare the application efficiency of LCV01 and LD05 in actual breeding, LD05 and LCV01 were crossed with Qixi5101 and DH382, respectively, and Qixi5101 and DH382 were used as recurrent parents for backcrossing. In each generation, positive plants were screened by sequentially using herbicide screening and PCR identification, which effectively avoided false-positive and false-negative results. From BC_1_F_1_ to BC_8_F_3_ in the Qixi5101 background, the transgenic positive rates of Qixi5101 (LD05) (33%, 61%, 36%, 51%, 43%, 64%, 34%, 31%, 26%, 17%) were significantly higher than that of Qixi5101 (LCV01) (15%, 23%, 31%, 17%, 19%, 25%, 19%, 22%, 5%, 4%) Figure 2(A, C). A similar trend was observed in the DH382 background, where the transgenic positive rates of DH382 (LD05) (40%, 27%, 63%, 46%, 27%, 61%, 63%, 53%, 21%, 27%) across BC_1_F_1_to BC_8_F_3_ were significantly higher than those of DH382 (LCV01) (18%, 21%, 23%, 23%, 24%, 26%, 21%, 20%, 5%, 7%) Figure 2(B, C). Based on these observed positive rates, the required screening population size could be reduced by 50%–75% when using the fusion gene strategy compared with the polygene polymerization strategy. By the BC_8_F_1_ generation, the background recovery rates of Qixi5101 (LD05), Qixi5101 (LCV01), DH382 (LD05), and DH382 (LCV01) reached 99.83 ± 0.17%, 99.82 ± 0.20%, 99.75 ± 0.28%, and 99.82 ± 0.22%, respectively, indicating that their genetic backgrounds were nearly identical to those of the respective recurrent parents Figure 2(D). LD05 not only offers a higher probability of identifying positive plants, but also reduces the screening workload and achieves a lower error rate. In summary, the fusion gene strategy exhibits significantly higher application efficiency in transgenic breeding than polygene polymerization, highlighting the great application value of LD05, which carries the *m2cryAb-vip3A* fusion gene.

**Figure 2.**
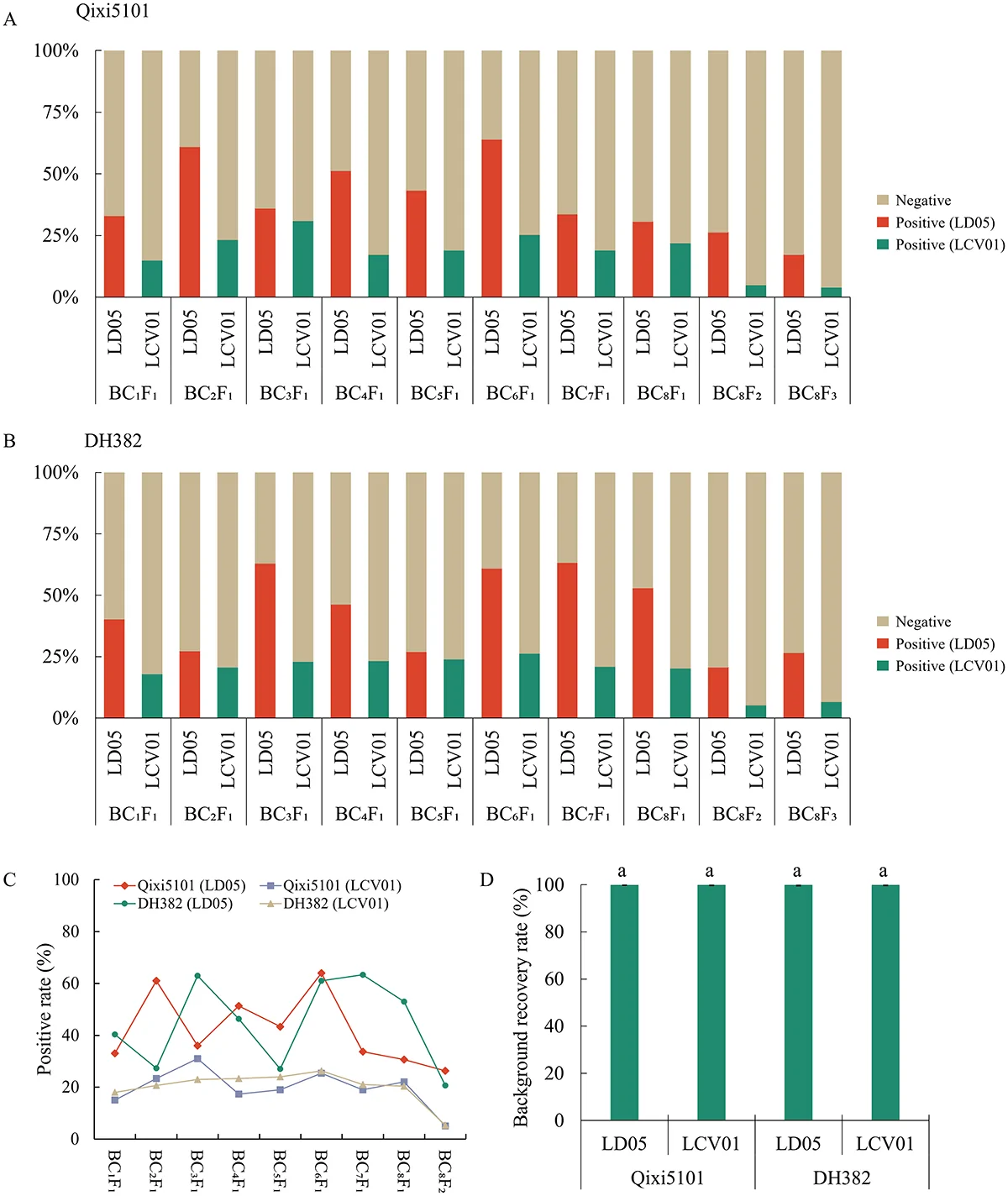
Application efficiency of fusion gene and polygene polymerization in transgenic breeding. A: actual transgenic positive rates from BC_1_F_1_ to BC_8_F_3_ in the Qixi5101 background. B: actual transgenic positive rates from BC_1_F_1_ to BC_8_F_3_ in the DH382 background. C: actual transgenic positive rates in each generation. D: background recovery rate at the BC_8_F_1_ generation. The same letter above the error bar indicates no significant difference in the background recovery rate between different materials (*p* > .05).

#### Identification of Insect-Resistant and Herbicide-Tolerant of Qixi5101 (LD05) and DH382 (LD05)

To evaluate the resistance of Qixi5101 (LD05) and DH382 (LD05) to *O. furnacalis*, *M. separata*, *H. armigera*, and *S. frugiperda* across three consecutive generations, leaves at the V5 stage were collected for laboratory bioassays against target pests. The results showed that Qixi5101 (LD05) and DH382 (LD05) exhibited significant and stable resistance to all four target pests Figure 3(A). In the leaves of Qixi5101 (LD05) and DH382 (LD05) across three consecutive generations, the corrected mortality rates of four pests were 100.00 ± 0.00% at 5 d after inoculation Figure 3(B, C). To evaluate field resistance, *O. furnacalis*, *M. separata*, *H. armigera*, and *S. frugiperda* were inoculated at the V5 and silking stages according to their natural occurrence. After inoculation, damage levels in Qixi 5101 (LD05) and DH382 (LD05) remained very low, whereas non‑GM controls showed high damage (7.41 ± 0.39–9.01 ± 0.39 at V5 stage; 6.76 ± 0.86–8.72 ± 0.86 at silking stage) Figure 3(D, E). For herbicide tolerance, gradient glufosinate doses were sprayed at V5. One week after treatment, all non‑GM plants died (100.00 ± 0.00% injury). At 1× and 2× doses, the GM lines showed no visible damage (0.00 ± 0.00% injury, 100.00 ± 0.00% survival). At 4× dose, they still achieved 100% survival, with only minor leaf chlorosis/curling (injury rates: 8.36 ± 2.01% and 11.42 ± 4.32%), which recovered within 4 weeks (Table S1). These results demonstrate that Qixi 5101 (LD05) and DH382 (LD05) possess strong resistance to major lepidopteran pests and high tolerance to glufosinate.

**Figure 3.**
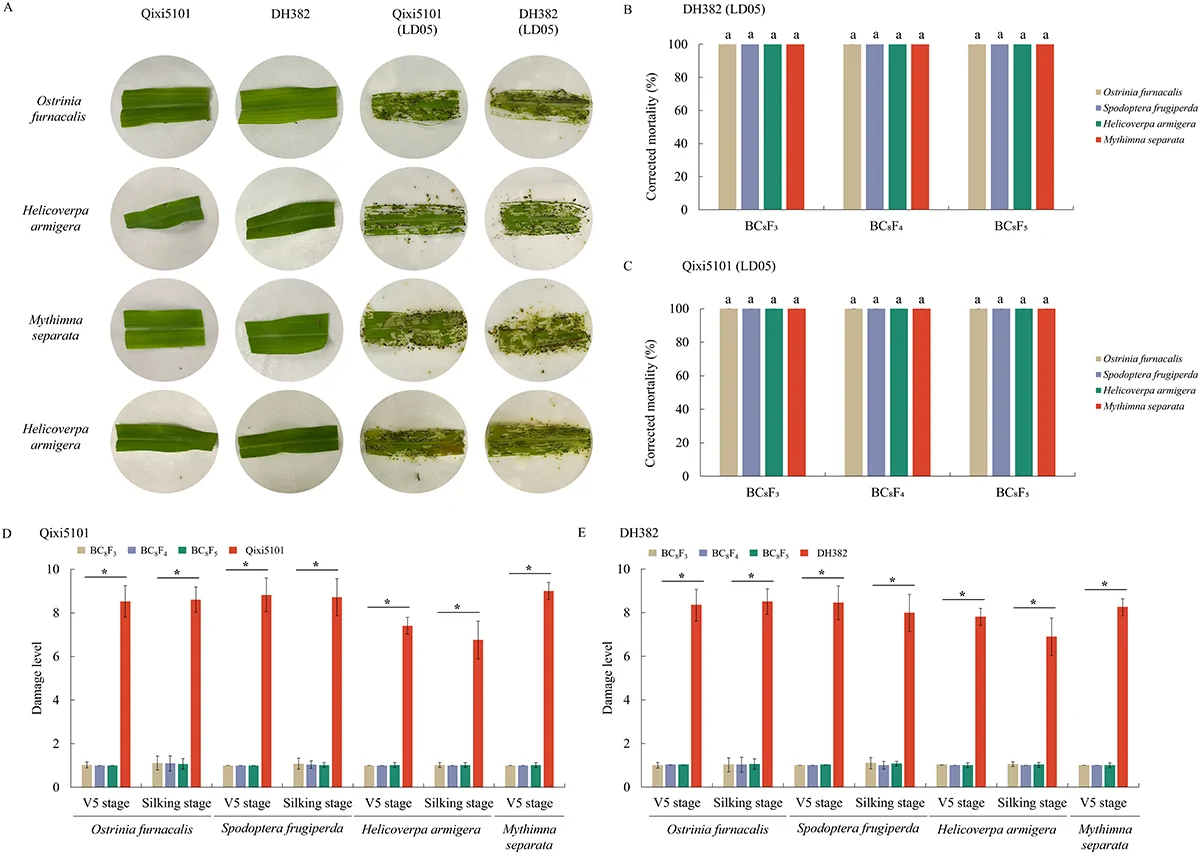
Insect resistance of Qixi5101 (LD05) and DH382 (LD05). A: resistance of the BC_8_F_3_ generation to target pests. B: corrected mortality rates of pests fed on leaves of Qixi5101 (LD05) across three generations. C: corrected mortality rates of pests fed on leaves of DH382 (LD05) across three generations. D: field insect resistance of Qixi5101 (LD05). E: field insect resistance of DH382 (LD05). Data are presented as mean ± standard error (*n* = 20 technical replicates per sample). The same letter above the error bars indicates no significant difference in corrected mortality (*p* > .05). Asterisks (*p* < .05) above the bars indicate significant differences in damage level.

#### Expression Analysis of Target Gene and Protein in Ludan509 (LD05) and Denghai605 (LD05)

To investigate whether the target genes are transcribed and translated in Ludan 509 (LD05) and Denghai 605 (LD05), the expression of the target genes (*m2cryAb-vip3A* and *bar*) and their corresponding proteins (M2CryAb-VIP3A and PAT) was examined in different tissues at different developmental stages. The highest expression levels were observed in V5 stage leaves (m2cryAb-vip3A: Ludan 509 (LD05) (33.97 ± 3.42)/Denghai 605 (LD05) (42.97 ± 2.70)), followed by stems and roots (m2cryAb-vip3A: Ludan 509 (LD05) (23.02 ± 2.34 and 16.26 ± 2.80)/Denghai 605 (LD05) (35.89 ± 2.80 and 27.52 ± 2.46)), moderate levels in kernels and silks (m2cryAb-vip3A: Ludan 509 (LD05) (16.95 ± 1.11 and 10.25 ± 2.10)/Denghai 605 (LD05) (18.20 ± 1.41 and 13.93 ± 4.20)), and the lowest levels in pollen and bracts (m2cryAb-vip3A: Ludan 509 (LD05) (6.40 ± 1.51 and 4.31 ± 1.38)/Denghai 605 (LD05) (6.46 ± 0.59 and 6.75 ± 1.04) Figure 4(A, E). The expression pattern of *bar* is highly consistent with *m2cryAb-vip3A* in the tissues of both hybrids Figure 4(B, F). The accumulation level of the target protein (M2CryAb-VIP3A and PAT) was highly consistent with its transcript abundance and exhibited tissue specificity Figure 4(C, D, G, H). No expression of the target gene or protein was detected in the Non-GM control. These results indicate that the target gene (*m2cryAb-vip3A* and *bar*) can be efficiently expressed and translated in Ludan 509 (LD05) and Denghai 605 (LD05), with stable expression.

**Figure 4.**
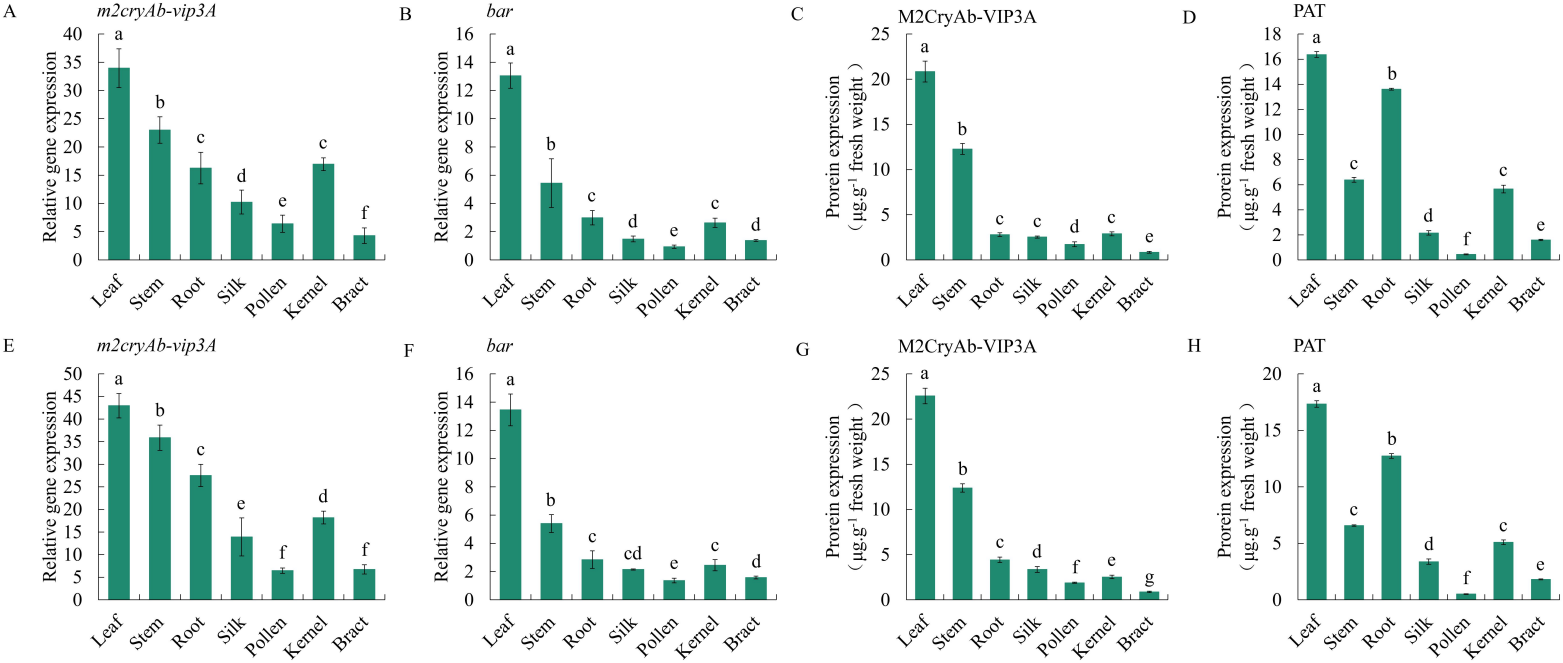
Expression analysis of target gene and protein in Ludan509 (LD05) and Denghai605 (LD05). A, E: tissue-specific expression of *m2cryAb-vip3A* in Ludan509 (LD05) and Denghai 605 (LD05). B, F: tissue-specific expression of *bar* in Ludan509 (LD05) and Denghai 605 (LD05). C, G: tissue-specific accumulation of M2CryAb-VIP3A in Ludan509 (LD05) and Denghai 605 (LD05). D, H: tissue-specific accumulation of pat in Ludan509 (LD05) and Denghai 605 (LD05). Means ± SD (*n* = 3 technical replicates per sample) are shown. Different letters indicate statistically significant differences in expression or accumulation levels across tissues (*p* < .05).

#### Identification of Insect-Resistant and Herbicide-Tolerant of Ludan509 (LD05) and Denghai605 (LD05)

Field trials showed that Ludan 509 (LD05) and Denghai 605 (LD05) exhibited high resistance to *O. furnacalis*, *S. frugiperda*, *H. armigera* (in heart leaves, female spikes, and straws) and to *M. separata* (in heart leaves), with damage levels of 1.00 ± 0.00–1.82 ± 0.06, significantly lower than non‑GM controls (>7.00). In bioassays, mortality rates of target pests on controls ranged from 10.24 ± 2.62% to 21.30 ± 5.40%, whereas those on GM hybrids exceeded 98.00% (Figure 5). For glufosinate tolerance, graded doses were sprayed at V5. One week after treatment, all non‑GM hybrids died (100.00 ± 0.00% injury). At 1× and 2× doses, GM hybrids showed no visible damage (0.00 ± 0.00% injury, 100.00 ± 0.00% survival). At 4× dose, they still achieved 100.00 ± 0.00% survival, with only minor leaf chlorosis/curling (injury rates: 12.35 ± 4.33% and 14.26 ± 3.76%), which fully recovered within 4 weeks (Table S2). These results demonstrate that Ludan 509 (LD05) and Denghai 605 (LD05) possess strong resistance to major lepidopteran pests and high tolerance to glufosinate.

**Figure 5.**
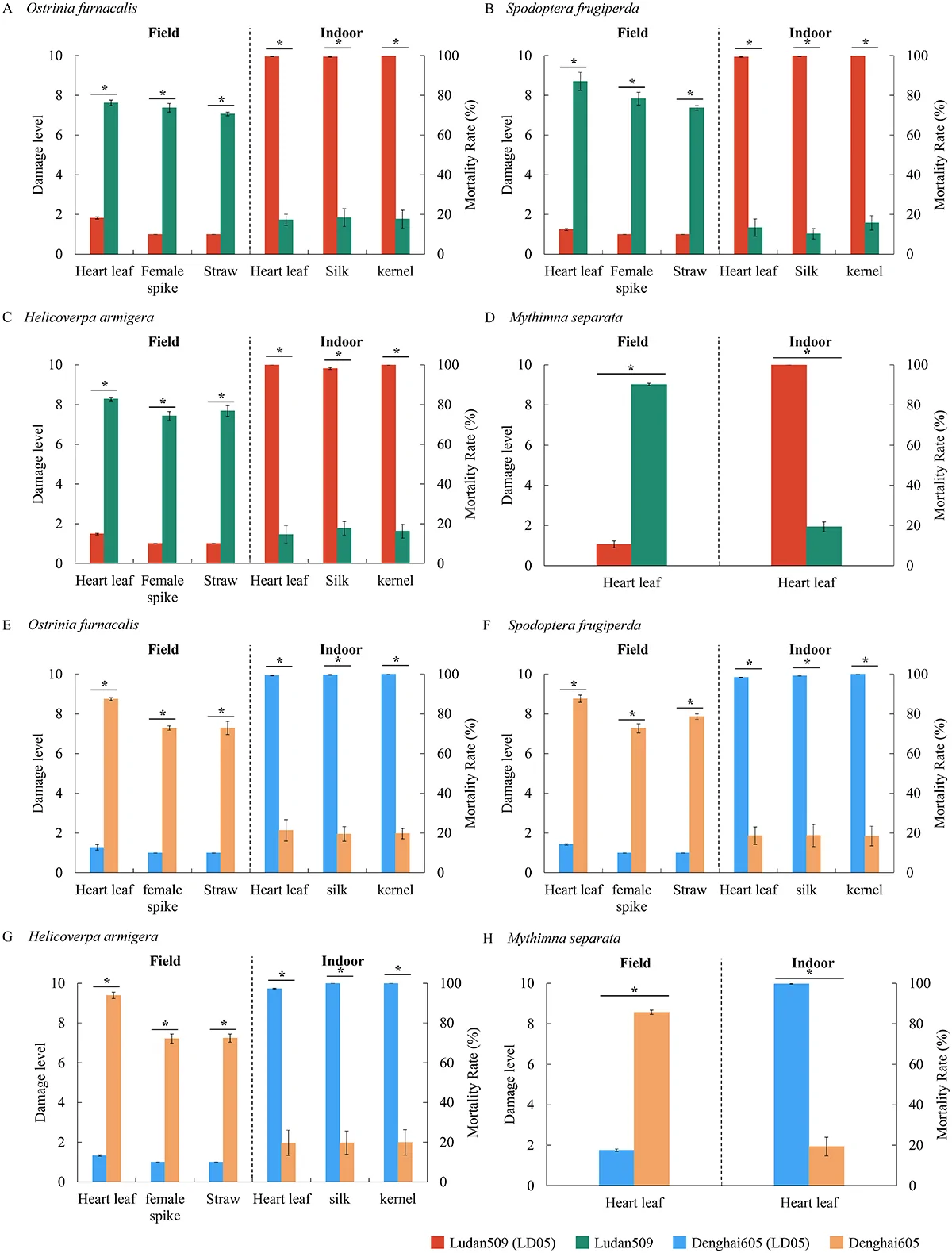
Insect resistance of Ludan509 (LD05) and Denghai605 (LD05). A-D: field insect resistance experiments and laboratory bioassays of Ludan509 (LD05). E-H: field insect resistance experiments and laboratory bioassays of Denghai605 (LD05). Data are presented as mean ± standard error (*n* = 3 replicates per treatment). Asterisks (*p* < .05) above the bars indicate significant differences between GM and non-GM.

#### Field Insect Resistance Performance of Ludan509 (LD05) and Denghai605 (LD05)

From 2023 to 2025, we evaluated the infestation dynamics of lepidopteran pests on Ludan509 (LD05) and Denghai605 (LD05) in Jinan and Hainan Figure 6(A). The pest pressure was relatively stable in both locations from 2023 to 2025. Pest infestation was more severe in Hainan than in Jinan. By the R5 stage, the infestation levels of non-GM Ludan509 in Jinan reached 25.77 ± 5.14%, 34.67 ± 6.34%, and 33.89 ± 2.03%, respectively, while those of non-GM Denghai605 reached 27.44 ± 6.84%, 34.89 ± 5.67%, and 34.56 ± 2.35%, respectively Figure 6(B-E). In Hainan, at the R5 stage, the infestation levels of non-GM Ludan509 were 74.00 ± 3.24%, 76.44 ± 5.15%, and 76.33 ± 2.96%, respectively, and those of non-GM Denghai605 were 73.11 ± 5.92%, 75.89 ± 3.18%, and 73.56 ± 4.62%, respectively Figure 6(F-I). The Ludan509 (LD05) and Denghai605 (LD05) exhibited very high insect resistance in both locations, with pest incidence below 7% throughout the growing period Figure 6(E, I). These results indicate that Ludan509 (LD05) and Denghai605 (LD05) maintained stable and excellent insect resistance across multiple years and locations.

**Figure 6.**
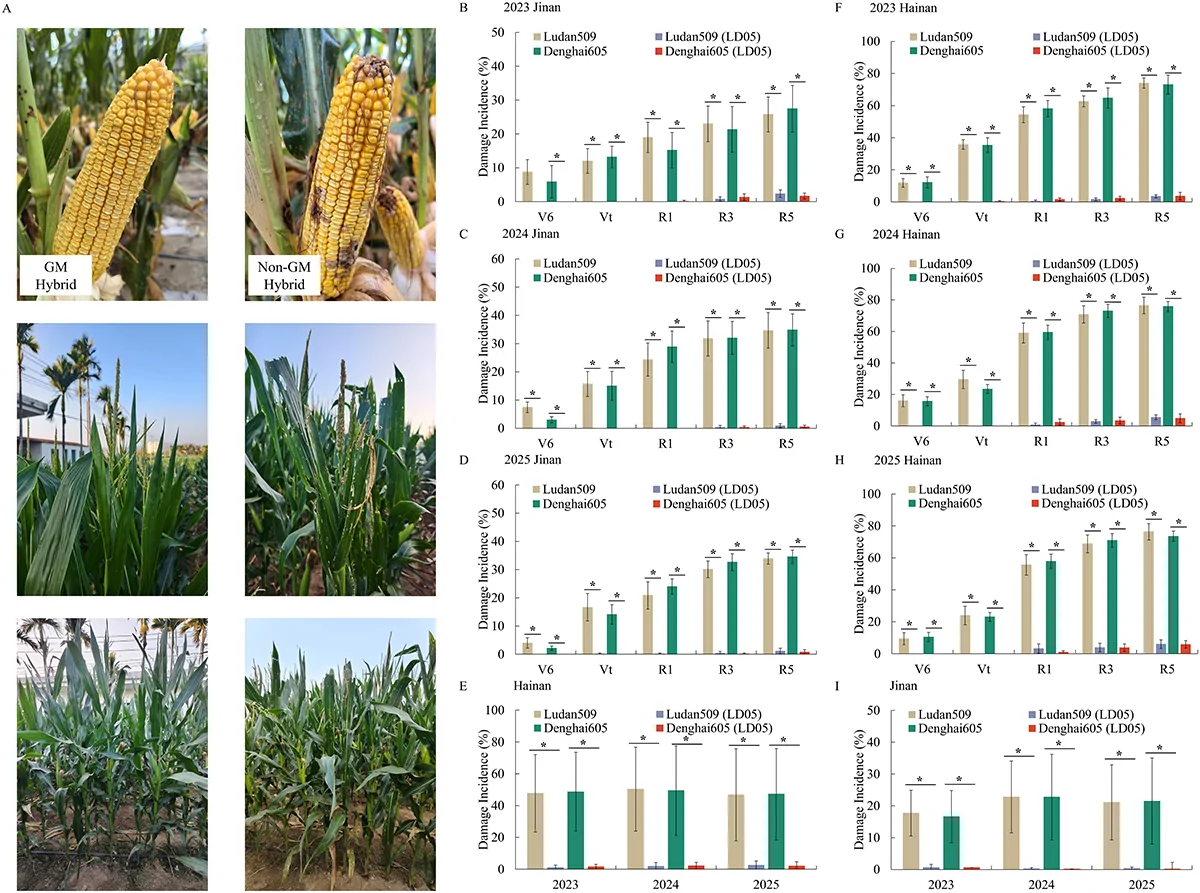
Insect infestation dynamics in GM and non-GM maize fields from 2023 to 2025. A: field insect resistance performance of the GM hybrids; B–D: pest infestation levels at different growth stages of maize in Jinan. E: total pest infestation levels in different years in Jinan. F–H: pest infestation levels at different growth stages of maize in Hainan. I: total pest infestation levels in different years in Hainan. Data are presented as mean ± standard error (*n* = 3 replicates per hybrid). Asterisks (*p* < .05) indicate significant differences.

#### Economic Benefits of GM Maize

From 2023 to 2025, under normal field management conditions, the yield of non-GM hybrids Ludan509 and Denghai605 was slightly lower than that of GM Ludan509 (LD05) and Denghai605 (LD05), but the difference was not significant Figure 7(A-D). In 2025, we calculated the field management costs for GM maize and non-GM maize. In Hainan, non-GM maize required five sprays of insecticides (US$558 ha^−1^) and three sprays of herbicides (US$56.35 ha^−1^) per season, with an associated spraying cost of US$85.5 ha^−1^. GM maize only needed to be sprayed with herbicide three times (US$27 ha^−1^), with an associated spraying cost of US$51.75 ha^−1^. GM maize cost US$621.1 ha^−1^ less than non-GM maize Figure 7(I, K, M, O). Because pest infestation in Jinan is lighter than in Hainan, insect spraying frequency in Jinan is lower Figure 7(E-H). GM maize costs US$432 ha^−1^ less than non-GM maize Figure 7(J–P). Under the condition of ensuring yield, the overall management cost of GM maize is reduced due to the elimination of insecticide, lower herbicide costs, and reduced application costs.

**Figure 7.**
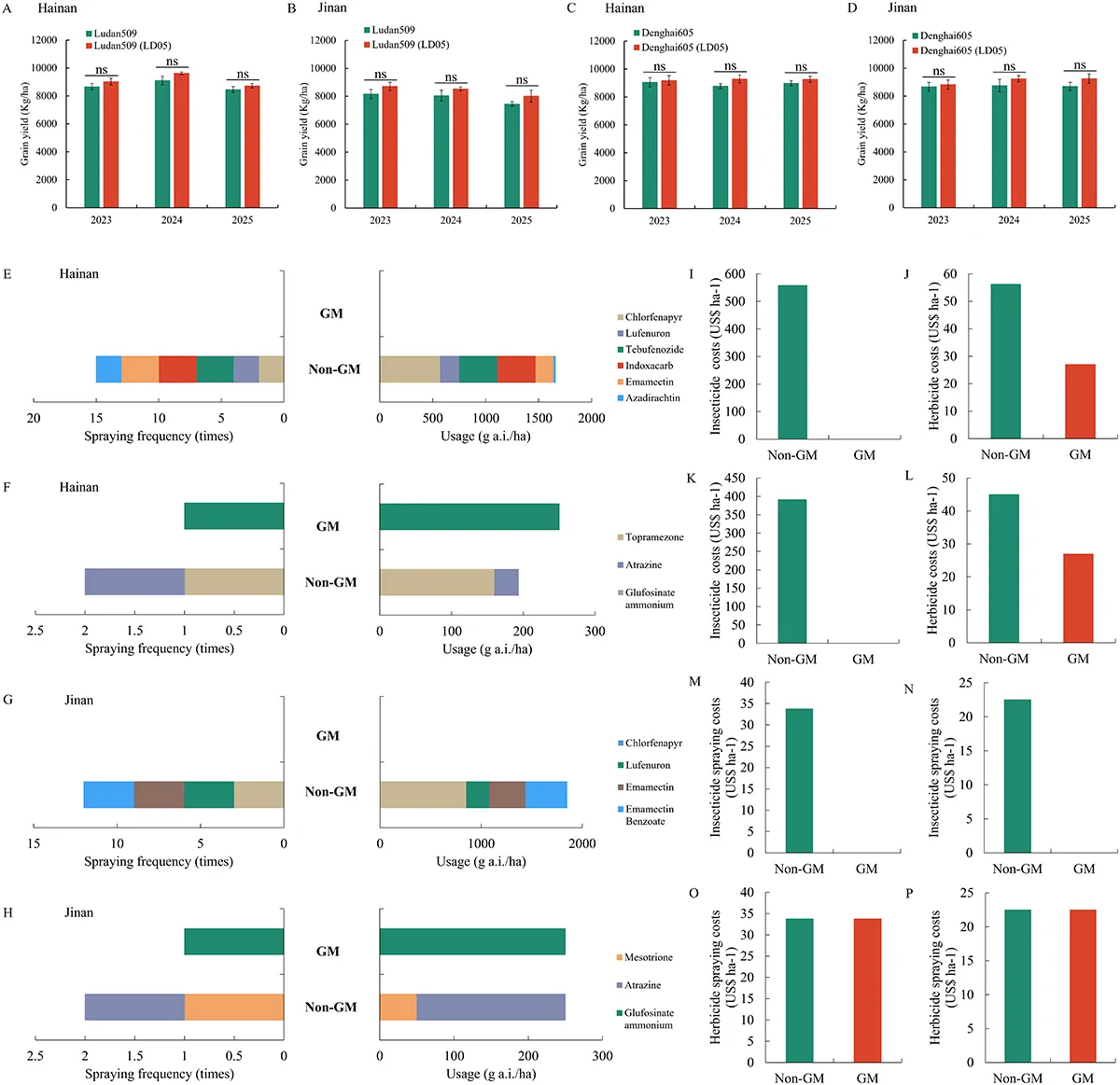
Economic benefits of GM maize. A-D maize yield in field trials in different years and locations. E-H: dosage and frequency of insecticide and herbicide applications in Hainan and Jinan. I, K, M, O: cost of insecticide and herbicide applications in Hainan. J, L, N, P: cost of insecticide and herbicide applications in Jinan. Grain yield for each hybrid was determined from three replicate plots. ns (*p* > .05) above the bars indicates no significant difference in yield.

## Conclusion

Cry proteins exhibit significant insecticidal activity against target lepidopteran pests such as *O. furnacalis*, but they are insensitive or show low sensitivity to pests like *S. frugiperda*, and long-term large-scale application has led to field resistance in some pest populations.^33,34^ Vip3 proteins have different modes of action and target receptors compared to Cry proteins, and their synergistic use can broaden the insecticidal spectrum and delay resistance evolution.^35,36^ Therefore, stacking *Cry* and *Vip3Aa* genes has become a core research direction for next‑generation insect‑resistant maize.^30,37,38^ In this study, LD05 (carrying the *m2cryAb-vip3A* fusion gene) and LCV01 (carrying two independent genes, *Cry1Ab* and *Vip3Aa*) exhibited significant resistance to four lepidopteran pests, including *Ostrinia furnacalis*, *Mythimna separata*, *Helicoverpa armigera*, and *Spodoptera frugiperda* (Figure 1). This broad-spectrum activity not only expanded the insecticidal spectrum but also reduced the likelihood that pests insensitive to only one of the proteins would survive.

Currently, the integration of multi‑gene insect resistance is mainly achieved through two technical approaches: polygene polymerization and fusion genes. The former, polygene polymerization, combines multiple independent genes into the same variety through crossing or multiple transformations. Although each gene is expressed independently, this approach suffers from problems such as a long breeding cycle, complex progeny segregation, high screening workload, genetic linkage drag, and high regulatory approval costs. The latter, the fusion gene approach, links the coding sequences of multiple insect‑resistant genes into a single open reading frame via a linker peptide, driven by a single promoter. It offers core advantages including a single transformation event, a single insertion site, and Mendelian inheritance at a single locus, thereby fundamentally simplifying the genetic regulation mechanism. This study compared the breeding efficiency of two transformation events, LD05 and LCV01, and confirmed that the fusion gene strategy offers significant efficiency advantages. In backcross generations, the positive rate of LD05 was consistently around 50% (compared to 25% for LCV01); in selfing generations, the homozygosity rate of LD05 reached 25% (compared to 6.25% for LCV01) Figure 2(A-C). In each generation, the actual positive rates of LD05 in both Qixi5101 and DH382 backgrounds were much higher than those of LCV01 Figure 2(D). This allows a 50%–75% reduction in the scale of the screening population, eliminates the need for multiplex PCR or separate detection of multiple loci, and significantly lowers the risks of “positive escape” and “false positives.” Moreover, the regulatory approval cost for a single transformation event is much lower than that for stacking multiple events, further enhancing the application value and commercial potential of LD05.

The stable multi-generational inheritance of target traits is a core prerequisite for the commercial application of GM crops. In LD05, the target genes and proteins were stably expressed and accumulated across multiple generations and maintained extremely high resistance to various lepidopteran pests over multiple generations.^30^ In this study, we systematically analyzed the insect resistance performance of LD05‑derived transgenic inbred lines across three consecutive generations. The GM inbred lines exhibited extremely high resistance to major lepidopteran pests: corrected mortality reached 100% in laboratory bioassays, and field damage levels at the V5 and silking stages were both below 1.5 (highly resistant), significantly outperforming non‑GM controls (damage > 6.5, susceptible) (Figure. 3). This also confirms that the transgenic inbred lines have high tolerance to glufosinate (Table S1). These results show that the LD05 event exhibits excellent phenotypic stability over multiple generations, fully meeting the technical requirements for commercial application.

The ultimate value of transgenic traits is reflected in the production and application of hybrids. Reducing planting costs and increasing income is a key driving factor for promoting the popularization and application of GM maize.^3,39,40^ In this study, Qixi5101 (LD05) and DH382 (LD05) were crossed with testers to generate GM hybrids Ludan509 (LD05) and Denghai605 (LD05). The *m2cryAb-vip3A* and *bar* genes were stably transcribed and translated in various tissues, with consistent expression levels and no evidence of transgene silencing, thereby confirming the structural integrity of the fusion gene expression cassette and the functional reliability of its regulatory elements (Figure 4). There are significant differences in the temporal and spatial expression of the *m2cryAb-vip3A* and *bar* genes, which may be attributable to the effects of maize variety, developmental stage, and tissue type.^41–43^ The median lethal concentration (LC50) represents the protein dose required to kill 50% of the tested larvae. The lower the value, the stronger the insecticidal activity. Previous studies have shown that the M2CryAb-VIP3A fusion protein had a relatively low LC50 of 24 ng cm^−2^ against *Spodoptera frugiperda* and that it not only caused larval mortality but also significantly inhibited the growth and development of *S. frugiperda* larvae.^27^ Under both field and laboratory conditions, damage ratings remained below 2.0 and corrected mortality exceeded 98%, indicating that the accumulation of the fusion protein was sufficient to rapidly kill four target lepidopteran pests. This high-dose expression pattern is consistent with the requirements of the high-dose/refuge strategy (Figure 5). Similarly, glufosinate tolerance exhibited a clear dose–response relationship: no visible injury was observed at 1× and 2× recommended medium doses, whereas the 4× dose caused only minor and transient leaf chlorosis/curling, followed by full recovery within 4 weeks (Table S2). These results indicate that PAT expression levels in Ludan 509 (LD05) and Denghai 605 (LD05) were sufficient to detoxify glufosinate even at four times the recommended field dose, further confirming the functional reliability of the herbicide-tolerance trait. To further elucidate the pest-specific responses to insecticidal proteins and their dose–response patterns, future studies could establish a broader range of M2CryAb-VIP3A dose gradients for different target pests and integrate transcriptomic, proteomic, and hormone/miRNA regulatory analyses to reveal pest-specific receptor binding, detoxification metabolism, and adaptation mechanisms, thereby providing a basis for resistance management and long-term durability assessment.^38,44^

Chemical control is currently the primary method for managing maize pests. However, long‑term use of chemical pesticides not only induces pest resistance but also disrupts the biotic communities in farmland.^45–48^ In actual field management, Ludan 509 (LD05) and Denghai 605 (LD05) do not require any chemical insecticides, thereby avoiding the negative impacts associated with chemical insecticide use. From 2023 to 2025, pest infestation was consistently more severe in Hainan than in Jinan. In Jinan, infestation levels were generally moderate, with *O. furnacalis*, *H. armigera*, and *Aphidoidea* as the dominant pests. Hainan experienced year‑round pest pressure, with *M. separata* as the overwhelmingly dominant pest, often accompanied by mixed infestations of *O. furnacalis*, *Spodoptera litura*, and other species. In this study, Ludan 509 (LD05) and Denghai 605 (LD05) maintained consistently low pest damage across 3 y and two locations, with no indication of increasing damage over time. This stability suggests that target lepidopteran pests did not develop detectable resistance to the fusion protein during the study period, which is consistent with the high-dose expression of M2CryAb-VIP3A and the dual mode of action of Cry1Ab and Vip3Aa. However, resistance development is a long-term evolutionary process, and continued monitoring across larger temporal and spatial scales is necessary to confirm the durability of resistance in Ludan 509 (LD05) and Denghai 605 (LD05) (Figure 6). Under the conditions tested in this study, the yields of the GM hybrids were slightly higher than those of the non-GM controls across the 3 y and two locations Figure 7(A-D). The GM hybrids also showed reduced pesticide input costs, with the largest reduction of US$621.1 ha^−1^ observed in Hainan, where pest pressure was highest Figure 7(E-P). These results suggest that exogenous gene insertion did not cause evident transgenic drag or yield penalty under the tested environments, although the scope of inference is limited by the relatively small number of locations and years. The combined insect and herbicide resistance not only enables coordinated pest and weed control but also greatly reduces chemical pesticide use, thereby lowering production costs, improving product safety, and protecting the farmland ecosystem. It fully reflects the production and application value of the insect‑resistant and herbicide‑tolerant maize LD05.

Long-term cultivation of GM maize may drive the evolution of pest resistance through selection pressure and behavioral adaptation, thereby reducing its insecticidal efficacy. By 2021, more than ten target pests, including *Spodoptera frugiperda* and *Ostrinia furnacalis*, had developed varying levels of resistance to different Bt proteins. The sustainable use of transgenic insect-resistant maize depends on effective resistance management, and the gene fusion strategy provides important support for this purpose.^49^ Transgenic maize LD05 carries the insect-resistant fusion gene *m2cryAb-vip3A*, which possesses both Cry1Ab and Vip3Aa protein activities, thereby not only broadening the insecticidal spectrum and enhancing insect resistance but also helping to delay the evolution of pest resistance. The primary strategy for managing transgenic resistance is the “high‑dose/refuge” approach.^50^ The high‑dose expression of *m2cryAb‑vip3A* in Ludan 509 (LD05) and Denghai 605 (LD05) supports the implementation of this strategy. During the practical cultivation of these hybrids, incorporating a refuge strategy tailored to the local agricultural environment can greatly delay resistance evolution.^51–54^ Although the biosafety of LD05 was systematically evaluated during its certificate approval in accordance with the regulations of the Ministry of Agriculture and Rural Affairs of China, covering non-target organisms and herbicide resistance management, its commercial deployment still requires continuous monitoring and management of gene flow risk, target pest resistance, secondary pest outbreaks, weed succession, and refuge strategy to ensure long-term ecological safety and sustainability. Fusion gene strategies (such as LD05) integrate multiple functional units into a single genetic module through “one‑time transformation and integrated design,” offering advantages in breeding efficiency, genetic stability, and ease of application. This method is driven by a single promoter, ensuring synchronous expression of multiple insecticidal proteins and avoiding stoichiometric imbalances common in polygene polymerization, thereby providing a better genetic solution for delaying pest resistance. This study presents the complete pre‑commercialization chain of GM maize – from LD05 transformation to hybrid validation – validating the high efficiency and reliability of the *m2cryAb‑vip3A* fusion gene and offering a simple, cost‑effective pathway for multi‑trait improvement. With the integration of synthetic biology and gene editing, this strategy is expected to extend to areas such as disease resistance, stress tolerance, and quality improvement, providing a practical and efficient option for the simultaneous improvement of multiple maize traits. However, it should be noted that the field performance and economic benefit results reported here were obtained from only two locations (Jinan and Sanya) over a 3-y period (2023–2025) and therefore cannot be directly generalized to other maize production regions with different pest pressures, weed communities, labor costs, or market conditions. In particular, economic benefits may fluctuate with pest infestation levels, herbicide prices, and the cost of labor for pesticide application. Therefore, broader multi-environment trials across major maize-producing areas, farmer-scale demonstrations, and sensitivity analyses of key economic parameters are still needed to more reliably evaluate the practical applicability of LD05 and to provide a scientific basis for its commercialization decisions.

## Supplementary Material

Supplementary materials.docx

## Data Availability

The data that support the findings of this article are contained within the article and its supporting information published online.
