# Supplementary material for: Analysis of breeding efficiency and application of insect-resistant and herbicide-tolerant maize LD05
Source: GM Crops Food. 2026 Nov 11;17(1):2753273. doi: 10.1080/21645698.2026.2753273 (PMC13761377; doi:10.1080/21645698.2026.2753273)
Supplement: Supplementary file 1 [file KGMC_A_2753273_SM6988.docx]

Supplementary Table 1 Glufosinate tolerance of Qixi 5101 (LD05) and DH382 (LD05) at the BC_8_F_5_ generation

| Material | Herbicide dosage | 1 WAT^†^ | | 2 WAT | | 4 WAT | |
| --- | --- | --- | --- | --- | --- | --- | --- |
|  |  | Survival (%) | Damage (%) | Survival (%) | Damage (%) | Survival (%) | Damage (%) |
| Qixi5101 (LD05) | Control | 100.00±0.00a^§^ | 0.00±0.00c | 100.00±0.00a | 0.00±0.00b | 100.00±0.00a | 0.00±0.00b |
|  | 1×^‡^ | 100.00±0.00a | 0.00±0.00c | 100.00±0.00a | 0.00±0.00b | 100.00±0.00a | 0.00±0.00b |
|  | 2× | 100.00±0.00a | 0.00±0.00c | 100.00±0.00a | 0.00±0.00b | 100.00±0.00a | 0.00±0.00b |
|  | 4× | 100.00±0.00a | 8.36±2.01b | 100.00±0.00a | 0.00±0.00b | 100.00±0.00a | 0.00±0.00b |
| Qixi5101 | Control | 100.00±0.00a | 0.00±0.00c | 100.00±0.00a | 0.00±0.00b | 100.00±0.00a | 0.00±0.00b |
|  | 1× | 0.00±0.00b | 100.00±0.00a | 0.00±0.00b | 100.00±0.00a | 0.00±0.00b | 100.00±0.00a |
|  | 2× | 0.00±0.00b | 100.00±0.00a | 0.00±0.00b | 100.00±0.00a | 0.00±0.00b | 100.00±0.00a |
|  | 4× | 0.00±0.00b | 100.00±0.00a | 0.00±0.00b | 100.00±0.00a | 0.00±0.00b | 100.00±0.00a |
| DH382 (LD05) | Control | 100.00±0.00a | 0.00±0.00c | 100.00±0.00a | 0.00±0.00b | 100.00±0.00a | 0.00±0.00b |
|  | 1× | 100.00±0.00a | 0.00±0.00c | 100.00±0.00a | 0.00±0.00b | 100.00±0.00a | 0.00±0.00b |
|  | 2× | 100.00±0.00a | 0.00±0.00c | 100.00±0.00a | 0.00±0.00b | 100.00±0.00a | 0.00±0.00b |
|  | 4× | 100.00±0.00a | 11.42±4.32b | 100.00±0.00a | 0.00±0.00b | 100.00±0.00a | 0.00±0.00b |
| DH382 | Control | 100.00±0.00a | 0.00±0.00c | 100.00±0.00a | 0.00±0.00b | 100.00±0.00a | 0.00±0.00b |
|  | 1× | 0.00±0.00b | 100.00±0.00a | 0.00±0.00b | 100.00±0.00a | 0.00±0.00b | 100.00±0.00a |
|  | 2× | 0.00±0.00b | 100.00±0.00a | 0.00±0.00b | 100.00±0.00a | 0.00±0.00b | 100.00±0.00a |
|  | 4× | 0.00±0.00 | 100.00±0.00a | 0.00±0.00b | 100.00±0.00a | 0.00±0.00b | 100.00±0.00a |

^†^ WAT: weeks after treatment.

^‡^ Different herbicide concentrations. 1×, 2×, 4×, the recommended medium dose of glufosinate.

^§^ Values are presented as mean ± standard error. Different letters indicate significant differences within the same row (*p* < 0.05).

Supplementary Table 2 Glufosinate tolerance of Ludan 509 (LD05) and Denghai 605 (LD05)

| Material | Herbicide dosage | 1 WAT^†^ | | 2 WAT | | 4 WAT | |
| --- | --- | --- | --- | --- | --- | --- | --- |
|  |  | Survival (%) | Damage (%) | Survival (%) | Damage (%) | Survival (%) | Damage (%) |
| Ludan509 (LD05) | Control | 100.00±0.00a^§^ | 0.00±0.00c | 100.00±0.00a | 0.00±0.00b | 100.00±0.00a | 0.00±0.00b |
|  | 1×^‡^ | 100.00±0.00a | 0.00±0.00c | 100.00±0.00a | 0.00±0.00b | 100.00±0.00a | 0.00±0.00b |
|  | 2× | 100.00±0.00a | 0.00±0.00c | 100.00±0.00a | 0.00±0.00b | 100.00±0.00a | 0.00±0.00b |
|  | 4× | 100.00±0.00a | 12.35±4.33b | 100.00±0.00a | 0.00±0.00b | 100.00±0.00a | 0.00±0.00b |
| Ludan509 | Control | 100.00±0.00a | 0.00±0.00c | 100.00±0.00a | 0.00±0.00b | 100.00±0.00a | 0.00±0.00b |
|  | 1× | 0.00±0.00b | 100.00±0.00a | 0.00±0.00b | 100.00±0.00a | 0.00±0.00b | 100.00±0.00a |
|  | 2× | 0.00±0.00b | 100.00±0.00a | 0.00±0.00b | 100.00±0.00a | 0.00±0.00b | 100.00±0.00a |
|  | 4× | 0.00±0.00b | 100.00±0.00a | 0.00±0.00b | 100.00±0.00a | 0.00±0.00b | 100.00±0.00a |
| Denghai605 (LD05) | Control | 100.00±0.00a | 0.00±0.00c | 100.00±0.00a | 0.00±0.00b | 100.00±0.00a | 0.00±0.00b |
|  | 1× | 100.00±0.00a | 0.00±0.00c | 100.00±0.00a | 0.00±0.00b | 100.00±0.00a | 0.00±0.00b |
|  | 2× | 100.00±0.00a | 0.00±0.00c | 100.00±0.00a | 0.00±0.00b | 100.00±0.00a | 0.00±0.00b |
|  | 4× | 100.00±0.00a | 14.26±3.76b | 100.00±0.00a | 0.00±0.00b | 100.00±0.00a | 0.00±0.00b |
| Denghai605 | Control | 100.00±0.00a | 0.00±0.00c | 100.00±0.00a | 0.00±0.00b | 100.00±0.00a | 0.00±0.00b |
|  | 1× | 0.00±0.00b | 100.00±0.00a | 0.00±0.00b | 100.00±0.00a | 0.00±0.00b | 100.00±0.00a |
|  | 2× | 0.00±0.00b | 100.00±0.00a | 0.00±0.00b | 100.00±0.00a | 0.00±0.00b | 100.00±0.00a |
|  | 4× | 0.00±0.00b | 100.00±0.00a | 0.00±0.00b | 100.00±0.00a | 0.00±0.00b | 100.00±0.00a |

^†^ WAT: week after treatment.

^‡^ Different herbicide concentrations. 1×, 2×, 4× the recommended medium dose.

^§^ Values are presented as mean ± standard error. Different letters indicate significant differences within the same row (*p* < 0.05).
